# Comparative Study on Dynamic Mechanical Behavior and Power-Law Versus Johnson–Cook Constitutive Models of Quenched 42CrMo Steel

**DOI:** 10.3390/ma19163474

**Published:** 2026-08-17

**Authors:** Bicheng Guo, Jiyao Li, Xinjie Yuan, Feng Jiang, Wenyu Zhang, Yajing Li, Shizhang Liu, Yingxu Lin, Zhilong Xu

**Affiliations:** 1 College of Marine Equipment and Mechanical Engineering, Jimei University, Xiamen 361000, China; 202411802015@jmu.edu.cn (J.L.); 202512855025@jmu.edu.cn (X.Y.); 202421361102@jmu.edu.cn (W.Z.); 202421361037@jmu.edu.cn (Y.L.); 202312855008@jmu.edu.cn (S.L.); zhilong.xu@163.com (Z.X.); 2 Engineering Research Center of Anti-Fatigue Manufacturing for Marine Equipment, Xiamen 361000, China; 3 Institute of Manufacturing Engineering, Huaqiao University, Xiamen 361021, China; linyingxu77@163.com; 4 Chengyi College, Jimei University, Xiamen 361000, China

**Keywords:** low-temperature quenched and tempered 42CrMo, constitutive model, SHPB, high strain rate, temperature softening, power-law model, Johnson–Cook model

## Abstract

This study systematically investigates the dynamic mechanical behavior and constitutive modeling of low-temperature quenched and tempered 42CrMo steel under high-strain-rate and high-temperature conditions. Dynamic compression tests were performed using a split Hopkinson pressure bar (SHPB) system over a strain rate range of 460–6450 s^−1^ and a temperature range of 25–800 °C. The results show that the flow stress of quenched 42CrMo steel exhibits significant strain hardening and temperature softening effects, while its strain rate sensitivity is observed to be relatively low, especially under ultra-high-strain-rate conditions. Based on the experimental data, both the Power-Law and Johnson–Cook constitutive models were established. A hardness-based temperature softening coefficient was introduced to convert the experimental stress–strain curves into isothermal stress–strain curves, thereby effectively decoupling the coupled effects of strain rate and temperature. Error analysis indicates that the Power-Law model yields an average fitting error of 1.98%, which is superior to that of the Johnson–Cook model (3.23%), suggesting that the Power-Law model is more suitable for describing the dynamic mechanical behavior of low-temperature quenched and tempered 42CrMo steel. The findings of this study provide a reliable constitutive basis for numerical simulations of low-temperature quenched and tempered 42CrMo steel under extreme thermomechanical coupling conditions, such as high-speed cutting and impact forming.

## 1. Introduction

Notably, 42CrMo steel is widely used in key load-bearing components such as large rolls, gears, and crankshafts due to its excellent strength, hardenability, and impact resistance [1,2,3,4]. In this study, rolls are taken as the research object. The roll blank is made of low-temperature quenched and tempered 42CrMo steel, and local quenching is required during the machining process. Therefore, two types of heat-treated 42CrMo steel, namely the quenched state and the tempered state, are present on the roll workpiece. This study focuses on low-temperature quenched and tempered 42CrMo steel. During cutting and plastic forming processes, the material is subjected to extremely high strain rates and severe temperature rises [5]. Therefore, accurately describing the dynamic mechanical behavior of low-temperature quenched and tempered 42CrMo steel under the above extreme conditions and establishing a precise constitutive model are of great engineering significance for optimizing process parameters, improving machining quality, and extending component service life [6].

The dynamic mechanical behavior of materials under high-temperature and high-strain-rate conditions typically exhibits the coupled effects of strain hardening, strain rate hardening, and temperature softening [7]. For 42CrMo steel, understanding the competition among these three effects and their influence on flow stress serves as the theoretical basis for establishing a reliable constitutive model [8]. In terms of experimental research, the split Hopkinson pressure bar (SHPB) technique has been widely used to characterize the dynamic mechanical properties of materials under high-strain-rate conditions [9]. Lee and Liu investigated the dynamic compressive deformation behavior of S50C medium-carbon steel using the SHPB technique over a strain rate range of 1.1 × 10^3^–5.5 × 10^3^ s^−1^ and a temperature range of 25–800 °C. It was found that flow stress increased with increasing strain rate but decreased with increasing temperature, and the temperature sensitivity parameter of the material increased with increasing strain rate [10]. Behrens et al. systematically studied the effects of mechanical and thermal conditions on the microstructure and mechanical properties of materials during thermomechanical processing. It was found that a fine-grained bainitic structure could be obtained through appropriate thermomechanical treatment, with the yield strength increased to 1174 MPa, the elongation after fracture reaching approximately 12.6%, and the impact absorbed energy reaching 58.5 J. Their study also showed that after rapid cooling, the forming of AISI 4140 could meet the requirements of the tempered state without additional heat treatment, which provides important reference value for understanding the dynamic mechanical behavior of quenched 42CrMo steel and optimizing its heat treatment process [11].

In the field of constitutive modeling, researchers have developed various phenomenological and physically based constitutive models for different materials [12,13,14]. The Johnson–Cook constitutive model (J-C model), proposed by Johnson and Cook in 1983, has become one of the most widely used phenomenological constitutive models due to its concise form and physically meaningful parameters [15,16]. Benedict Stampfer and Germán González systematically calibrated the J-C constitutive parameters of AISI 4140 steel under two different tempering conditions. It was found that a single set of parameters could not accurately describe mechanical behavior under different initial hardness states, which highlights the necessity of parameter calibration for specific heat treatment states [17]. Samantaray et al. systematically compared the prediction performance of the J-C model, the modified Z-A model, and the Arrhenius-type constitutive model for the high-temperature flow behavior of 9Cr-1Mo steel. It was found that the physically based Z-A model performed the best under high-temperature and low-strain-rate conditions, whereas the J-C model exhibited lower prediction accuracy under these conditions due to its failure to consider the coupling effects between strain and temperature as well as between strain rate and temperature [18]. The Power-Law constitutive model (P-L model) has also been widely used to describe the dynamic mechanical behavior of metallic materials [19]. This model describes strain hardening, strain rate hardening, and temperature softening effects in a multiplicative form and is applicable over a wide range of strain rates and temperatures [20]. Zhu et al. conducted a comparative evaluation of constitutive models for orthogonal cutting simulation based on a coupled Eulerian–Lagrangian formulation. The results showed that the model incorporating strain-rate hardening and thermal softening exhibited higher accuracy in predicting cutting forces and chip morphology [21].

Although numerous studies have been conducted on the constitutive models of 42CrMo steel [22,23,24], and our previous work has partially addressed tempered 42CrMo steel and its constitutive models [25,26,27], comparative investigations on constitutive models for quenched 42CrMo steel under coupled high-strain-rate and high-temperature conditions remain relatively limited. In particular, a systematic evaluation of the fitting accuracy and applicable scope of different constitutive models for quenched 42CrMo steel is still lacking [28]. Moreover, existing studies typically calibrate constitutive models directly using the experimentally measured “adiabatic” stress–strain curves obtained from SHPB tests. However, during high-strain-rate deformation, the adiabatic temperature rise caused by the conversion of plastic work into heat leads to a decrease in flow stress, which couples the strain rate hardening effect with the temperature softening effect in the experimental data. Consequently, this coupling compromises the accuracy of parameter identification in constitutive model calibration.

To address the aforementioned research gap, this paper proposes to employ high-temperature Vickers hardness testing to obtain the thermal softening coefficient of the material, thereby substituting the thermal softening information traditionally derived from high-temperature SHPB experiments. The fundamental rationale of this method lies in the fact that hardness testing is a quasi-static process that does not involve strain rate effects; hence, the thermal softening coefficient obtained can reflect the intrinsic temperature-dependent softening characteristics of the material, independent of strain rate. By using this coefficient to perform isothermal correction on the true stress–true strain curves measured by SHPB experiments, the coupled influence of strain rate hardening and temperature softening can be effectively decoupled, thus enhancing the accuracy of constitutive model parameter calibration. This method has been preliminarily validated in our research team’s previous studies on TC4-DT titanium alloy [29].

Against this background, in the present study, dynamic compression experiments were conducted on quenched 42CrMo steel using the SHPB technique over a strain rate range of 460–6450 s^−1^ and a temperature range of 25–800 °C, and the dynamic mechanical behavior characteristics were systematically analyzed. Based on the experimental data, both the P-L model and the J-C model were established. The hardness-based thermal softening coefficient was introduced for isothermal correction to decouple the coupled effects of strain rate and temperature, and the fitting accuracies of the two models were compared through error analysis. This study aims to provide a more accurate constitutive basis for numerical simulations of low-temperature quenched and tempered 42CrMo steel in engineering applications such as high-speed machining and impact forming and also to offer an effective methodological reference for establishing isothermal constitutive models of metallic materials under coupled thermomechanical loading conditions.

## 2. Materials and Methods

### 2.1. Materials

**Figure 1:**
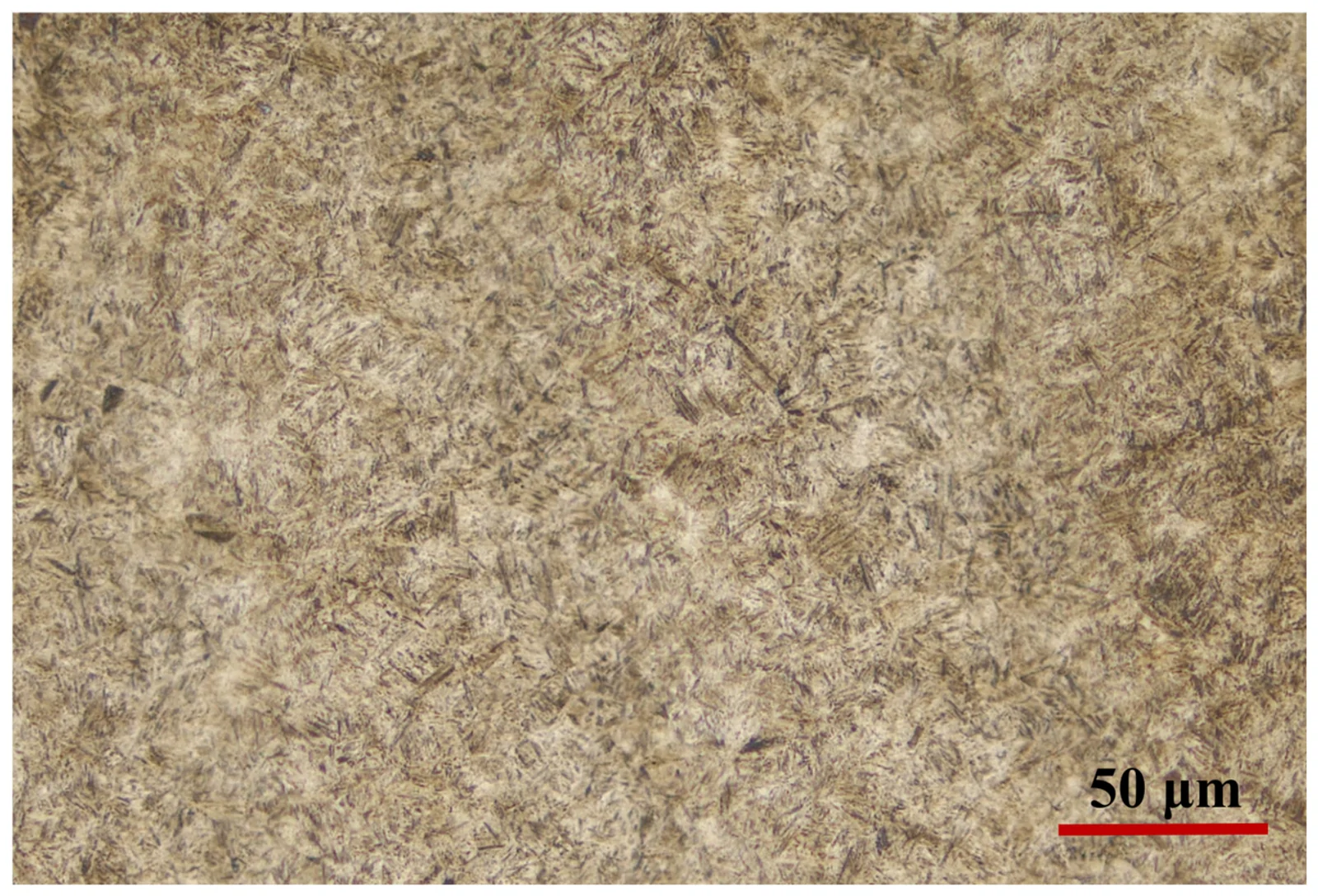
Microstructure of quenched 42CrMo steel.

**Figure 2:**
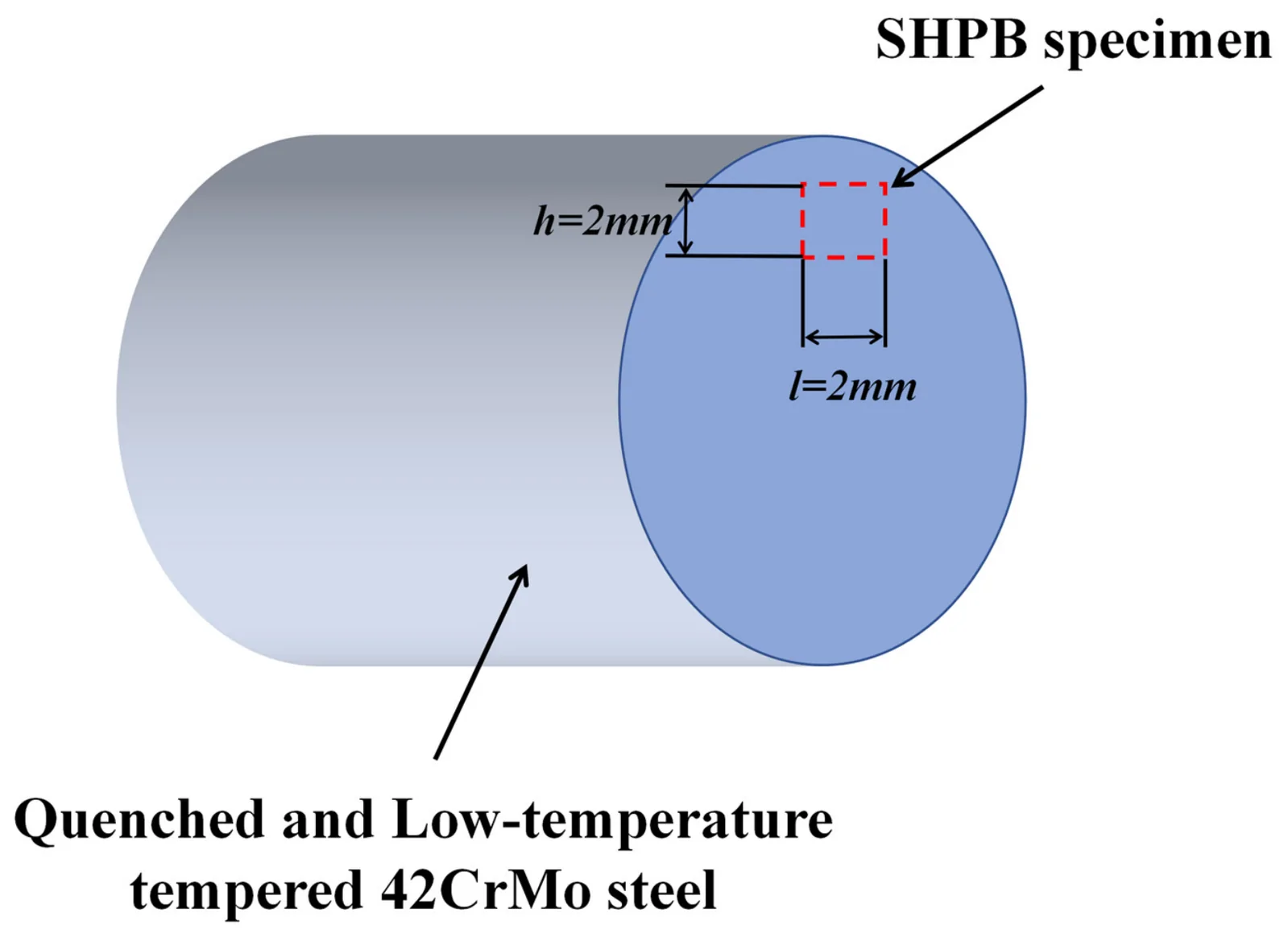
A schematic illustration of the sampling positions and dimensions of the SHPB specimens.

In this study, rolls are taken as the research object, and the commonly used material for rolls is 42CrMo steel. The heat treatment process for low-temperature quenched and tempered 42CrMo steel (hereinafter referred to as quenched 42CrMo steel) is as follows: the samples were heated to 860 °C and held for 1 h, then held at 250 °C for 1.5 h, and finally oil-cooled to room temperature. The heating rate was 10–13 °C/min. Oil quenching was employed, with a cooling rate of approximately 30 °C/s. The chemical composition is shown in Table 1, and the basic mechanical properties are presented in Table 2 [30]. Figure 1 shows the initial microstructure of 42CrMo steel after quenching and low-temperature tempering. A typical tempered martensite lath structure can be observed, with the microstructure being uniform and fine. Subsequently, the heat-treated specimens were machined to prepare 2 mm thick square block specimens, as shown in Figure 2.

### 2.2. Methods

**Figure 3:**
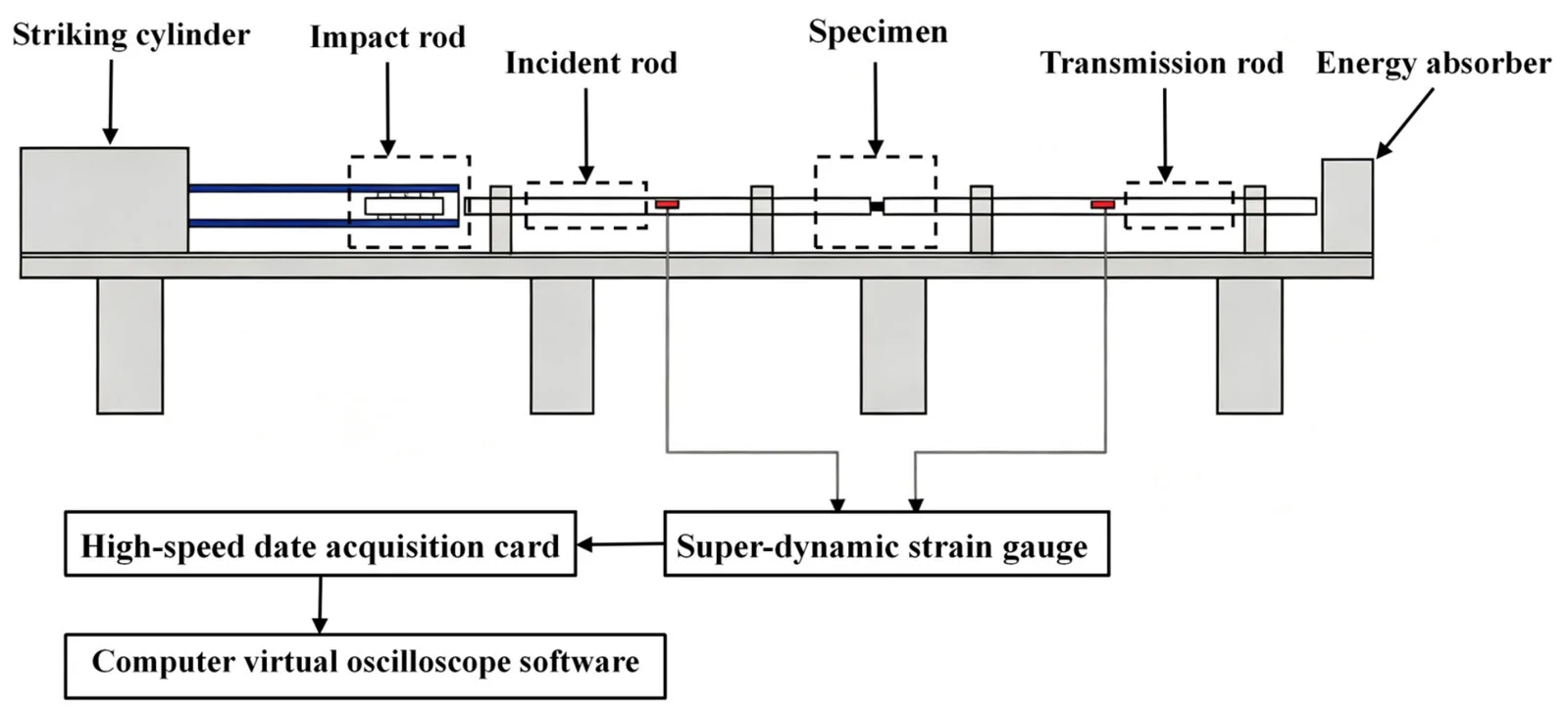
A schematic diagram of the Hopkinson pressure bar test system.

The SHPB technique was utilized to measure the mechanical properties of materials under high strain rates. The experimental apparatus, sourced from the School of Aeronautics at Northwestern Polytechnical University in China, is illustrated in Figure 3. The experiment was conducted using a standard Φ5 mm SHPB system, comprising 18Ni maraging steel bars with a diameter of 5 mm, an elastic modulus of 190 GPa, and a longitudinal wave velocity of 4900 m/s. Strain gauges were attached to the mid-span positions of the incident bar and the transmission bar. The voltage signals were recorded by an ultra-dynamic strain gauge (model SDY2107B) and a 40 MHz high-speed data acquisition card (model USB12047) with 12-bit resolution. The strain values were converted from the output voltage of 0.6 V according to the calibration range set at 2000 *μ*_ε_. Based on the fundamental principles of one-dimensional stress wave theory, the pulse duration generated upon impact is determined by the length of the striker bar. Upon collision between the striker bar and the incident bar, compressive stress pulses are generated simultaneously in both bars and propagate toward their respective opposite ends. Once the compressive pulse in the striker bar is reflected at the free end, it transforms into a tensile unloading wave that travels back toward the impact interface. Consequently, the pulse duration within the incident bar is precisely twice the length of the striker bar.

When the stress pulse propagating down the incident bar reaches the interface with the specimen, the impedance mismatch between the bar and the sample causes a portion of the pulse to be reflected back into the incident bar, forming the reflected wave. The remaining portion traverses the specimen and enters the transmission bar, generating the transmitted wave. The shapes and amplitudes of both the reflected and transmitted waves are intrinsically governed by the constitutive behavior of the specimen material. Strain gauges firmly mounted on the incident and transmission bars are capable of recording the complete waveforms of these reflected and transmitted pulses. Based on one-dimensional stress wave theory, the stress–strain relationship of the specimen material can be derived, under the critical assumption that the specimen maintains a state of stress equilibrium throughout the high-rate impact process. Ultimately, the temporal evolutions of the average stress *σ*(*t*), strain *ε*(*t*), and strain rate *έ*(*t*) are obtained and expressed as presented in Equations (1)–(3), respectively.

(1) $$\sigma (t)=EA\varepsilon_{T}(t)/A_{s}$$

(2) $$\varepsilon (t)=-\frac{2C_{0}}{L_{0}}\int_{0}^{t}\varepsilon_{R}(t)dt$$

(3) $$\dot{\varepsilon }(t)=-2C_{0}\varepsilon_{R}(t)/L$$

where *E* is the elastic modulus of the bar, *A* is the cross-sectional area of the bar, As is the cross-sectional area of the specimen, *C*_0_ is the one-dimensional elastic wave velocity of the bar, *C*_0_ = (*E*/*ρ*_o_)^1/2^, *ρ*_o_ is the density of the bar, and *L* is the length of the specimen. *ε*_R_ is the amplitude of the reflected strain wave, and *ε*_T_ is the amplitude of the transmitted strain wave.

From Equations (1)–(3), the relations between engineering stress, engineering strain, true stress, and true strain are obtained, as shown in Equations (4) and (5).

(4) $$\sigma_{T}=(1-\varepsilon (t))\sigma (t)$$

(5) $$\varepsilon_{T}=-\ln(1-\varepsilon (t))$$

As presented in Table 3, a series of tests at different strain rates and temperatures was conducted to characterize the dynamic mechanical response of 42CrMo steel under diverse conditions. In the experimental design, at least three repeated tests were conducted for each testing condition, and the average values of the results were used for subsequent analyses.

## 3. Results

### 3.1. Results of Tests of Mechanical Properties

**Figure 4:**
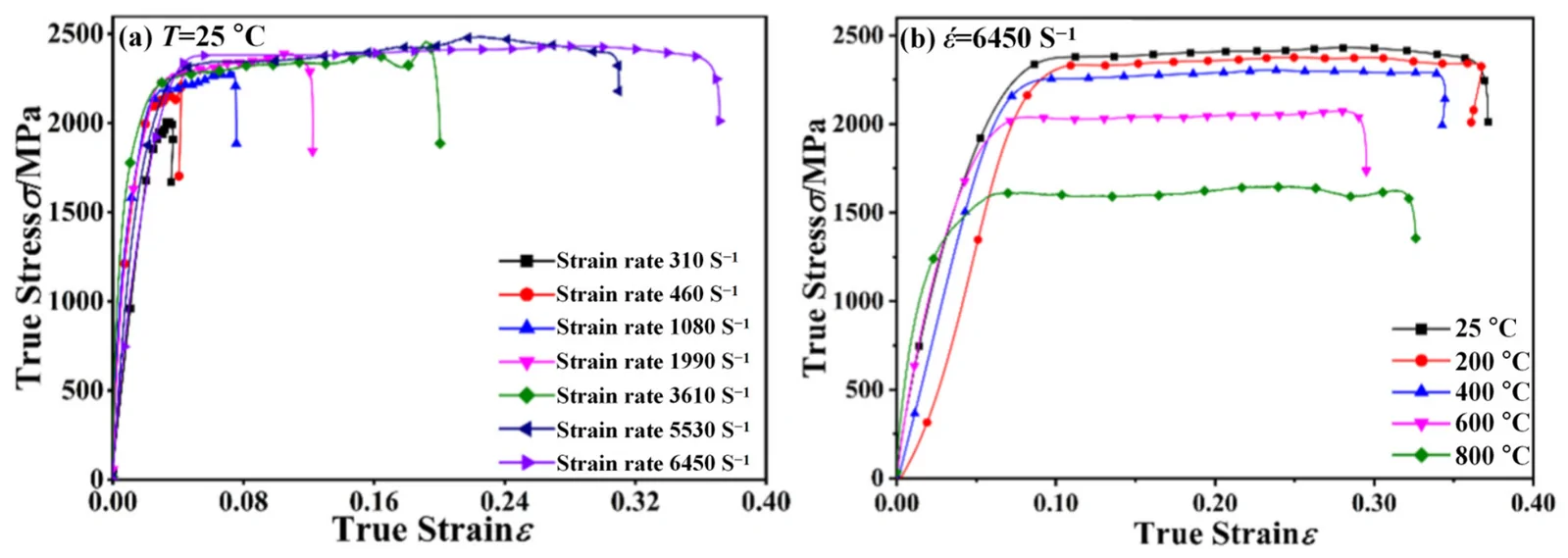
Stress–strain curves: (**a**) at different strain rates; (**b**) at different temperatures.

Quenched 42CrMo steel exhibits significant strain hardening characteristics during the plastic deformation stage, while its flow stress is significantly affected by the temperature softening effect. As shown in Figure 4a, at different strain rates, the true stress first increases with increasing true strain and then gradually stabilizes, indicating that the material has entered the plastic deformation stage. As shown in Figure 4b, at the same strain, the true stress decreases as the temperature increases. The strain hardening characteristic is attributed to the occurrence of intragranular slip during plastic deformation, which leads to a significant increase in dislocation density and lattice distortion. This reduces the plastic deformation capacity of the material while significantly enhancing its tensile strength, yield strength, and surface hardness. The decrease in true stress with increasing temperature is caused by the temperature softening effect. This evolution law is consistent with existing research results. Sui et al. also observed a similar trend of stress stabilization due to a significant increase in dislocation density through the analysis of the cyclic deformation behavior of 42CrMo steel under different heat treatment states. Previous studies have shown that temperature softening is commonly observed in 42CrMo steel during high-temperature deformation [31].

**Figure 5:**
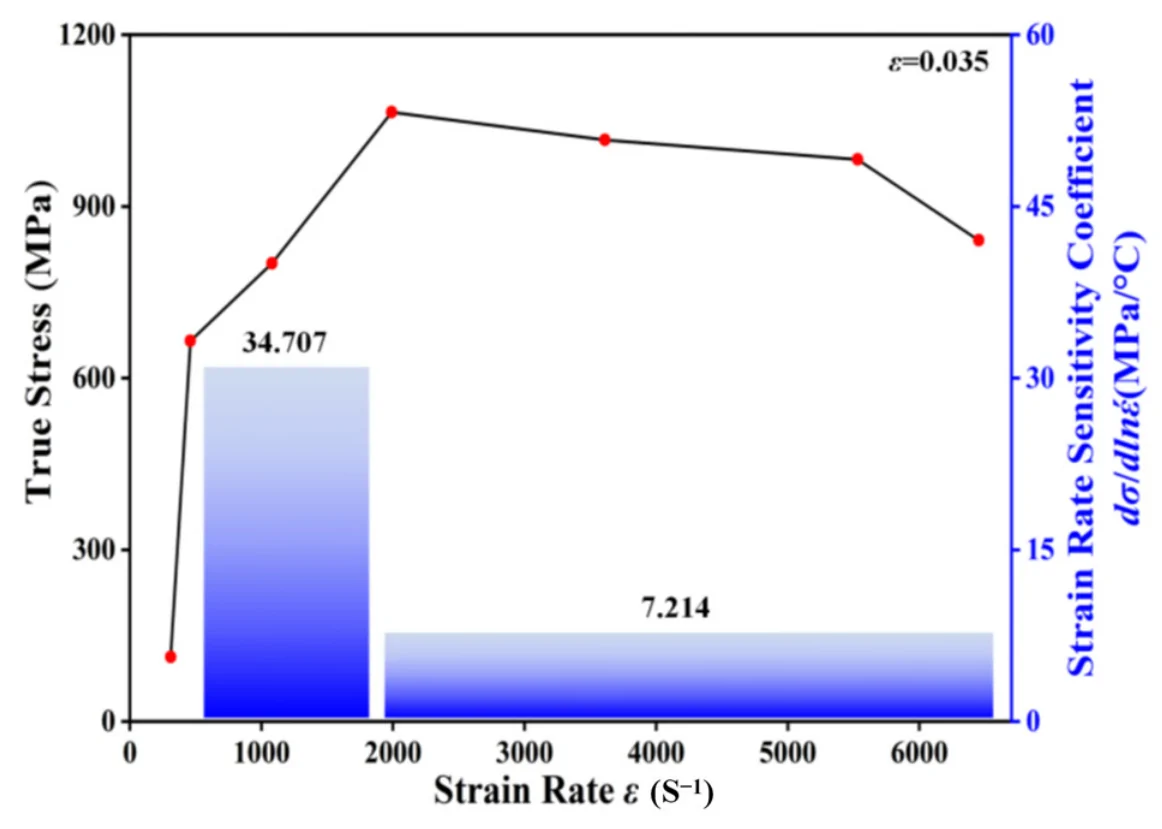
Variation in true stress with strain rate and strain rate sensitivity coefficient.

**Figure 6:**
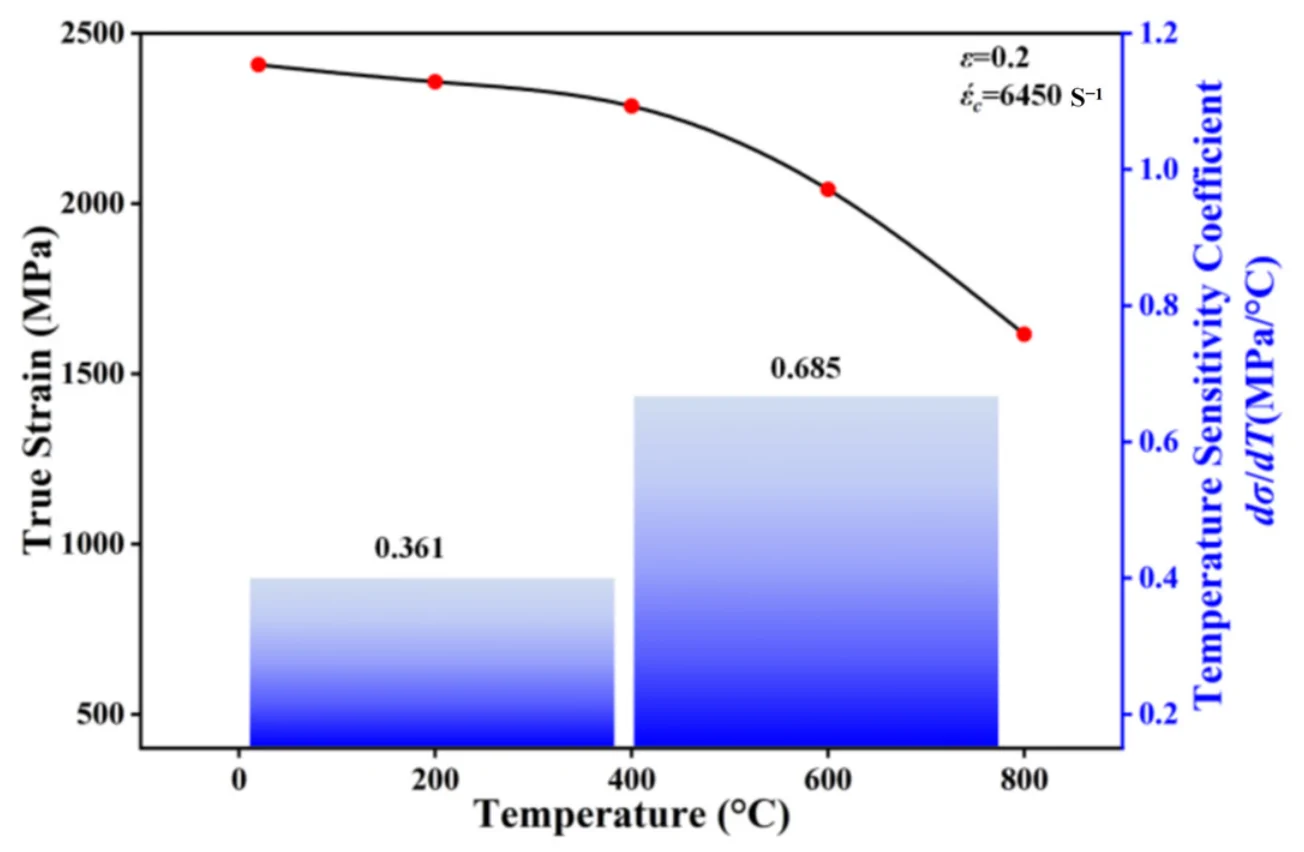
Variation in true stress with temperature and temperature sensitivity coefficient.

The strain rate sensitivity of quenched 42CrMo steel exhibits a critical strain rate of 1990 s^−1^, and its temperature sensitivity increases with increasing temperature. As shown in Figure 5, the true stress increases significantly when the strain rate is between 460 and 1990 s^−1^, while the change in true stress is not obvious and even shows a decreasing trend when the strain rate is between 1990 and 6450 s^−1^. The strain rate sensitivity coefficients for the strain rate ranges of 460–1990 s^−1^ and 1990–6450 s^−1^ are 34.707 and 7.214, respectively. As shown in Figure 6, at the same strain rate and strain level, the true stress decreases with increasing temperature, and the decreasing rate accelerates. The temperature sensitivity coefficients for the temperature ranges of 25–400 °C and 400–800 °C are 0.361 MPa/°C and 0.685 MPa/°C, respectively. The experimental results show that quenched 42CrMo steel has a low strain rate sensitivity, i.e., its mechanical properties exhibit significant insensitivity under high strain rate loading. The higher the temperature, the higher the temperature sensitivity coefficient, while the material is insensitive to temperature at lower temperatures [32]. In summary, quenched 42CrMo steel exhibits both strain rate sensitivity and temperature sensitivity during deformation. The strain rate sensitivity and temperature sensitivity of the material are affected by the strain rate and temperature: strain rate sensitivity decreases during high-strain-rate deformation, while temperature sensitivity increases during high-temperature deformation.

### 3.2. Adiabatic Temperature Rise

**Figure 7:**
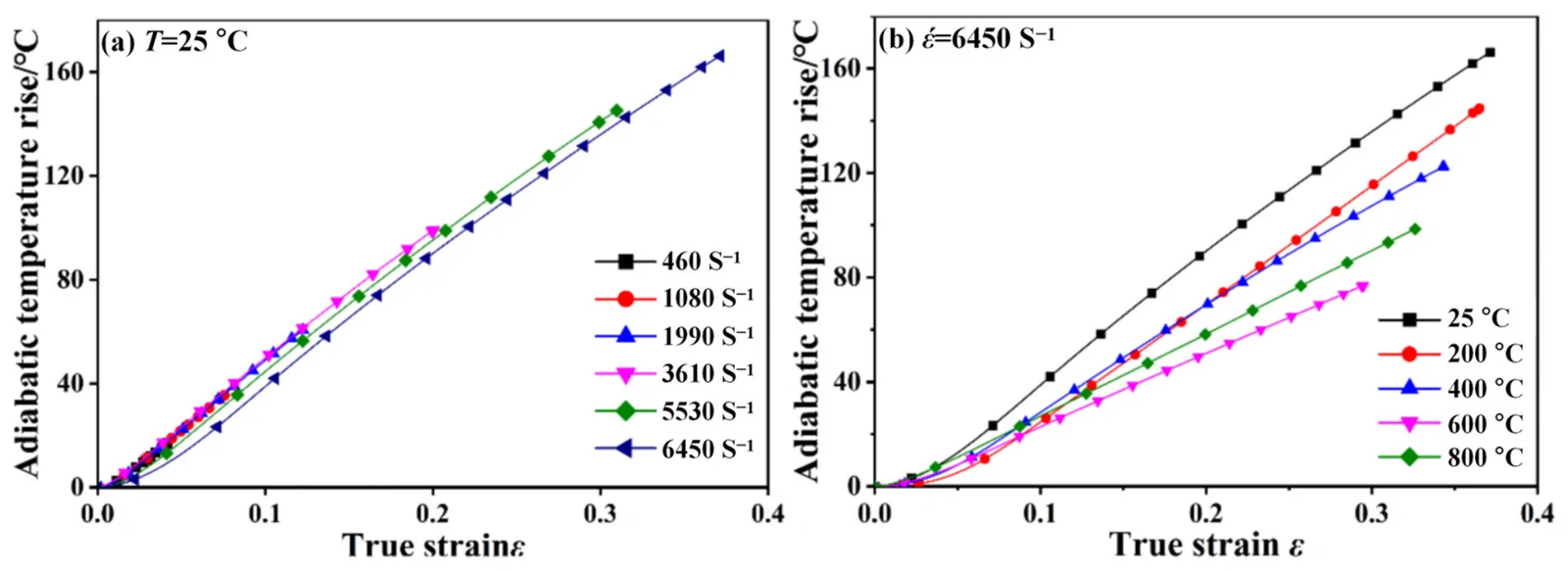
True strain and adiabatic temperature rise: (**a**) at different strain rates; (**b**) at different temperatures.

When the strain rate of dynamic deformation exceeds 10  s^−1^, the deformation process can be regarded as an adiabatic process, and the adiabatic temperature rise varies regularly with deformation conditions. The adiabatic temperature rise during dynamic deformation can be calculated using Equation (6). Figure 7a,b show the adiabatic temperature rise results under different conditions. It can be seen from this figure that as the strain rate increases, the strain increases, and the adiabatic temperature rise also increases, with a consistent rate of increase. At the same strain rate, as the temperature increases, the adiabatic temperature rise decreases, and its rate of increase also decreases. The above results indicate that the adiabatic temperature rise is jointly regulated by the strain rate and the initial temperature—a high strain rate promotes the increase in temperature rise, whereas a high initial temperature suppresses the temperature rise and its rate of increase.

(6) $$\Delta T=\frac{\beta }{\rho c}\int_{0}^{\varepsilon }\sigma d\varepsilon$$

where Δ*T* is the adiabatic temperature rise, *ρ* is the density of the material, *c* is the specific heat capacity of the material, *σ* is the stress of the material, *ε* is the strain of the material, and *β* is the energy conversion rate. It is generally assumed that all plastic work of the material is converted into adiabatic temperature rise, so *β* = 1.

### 3.3. Isothermal Stress–Strain Curves

**Figure 8:**
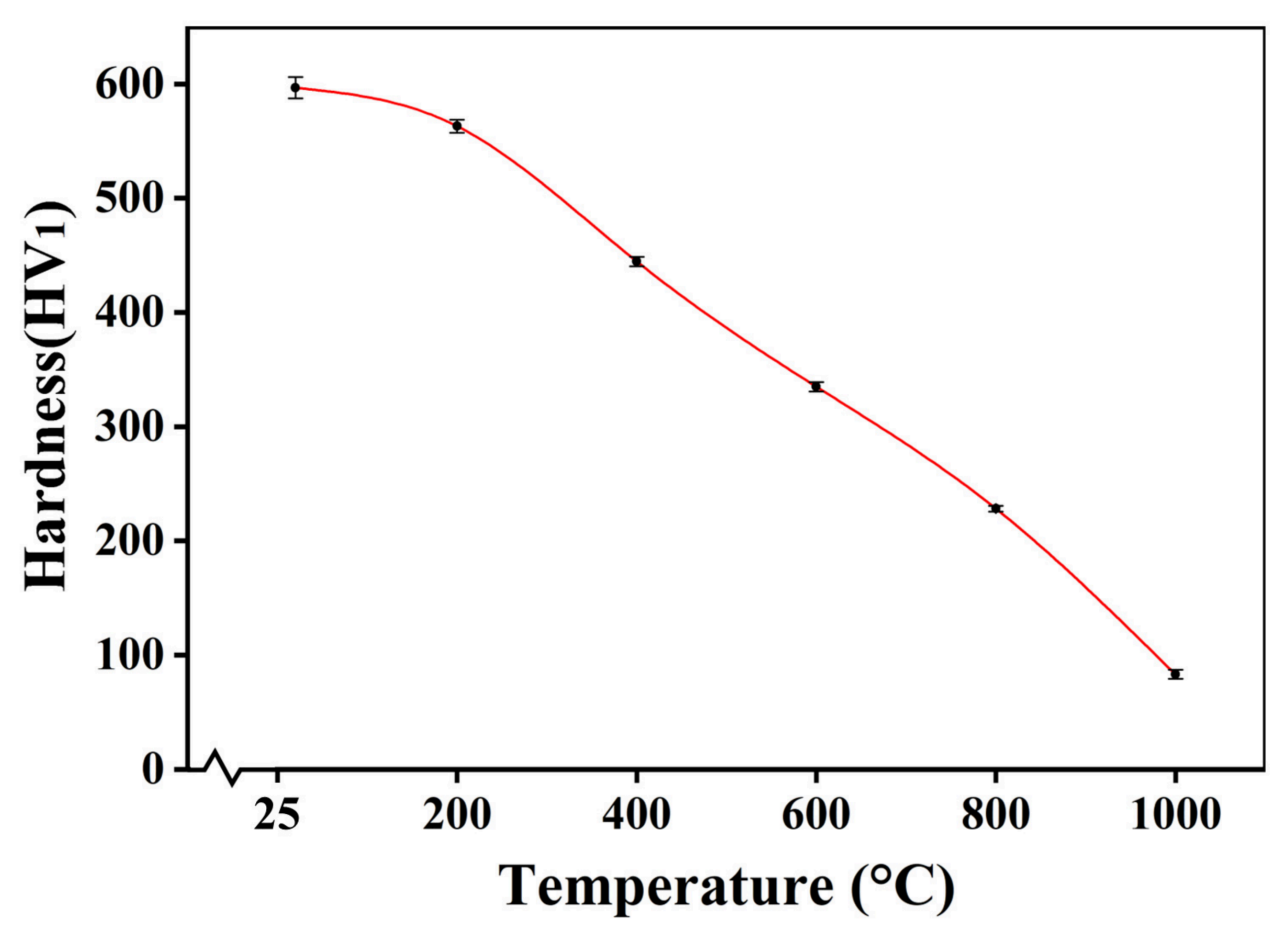
Vickers hardness HV_1_ at different temperatures.

**Figure 9:**
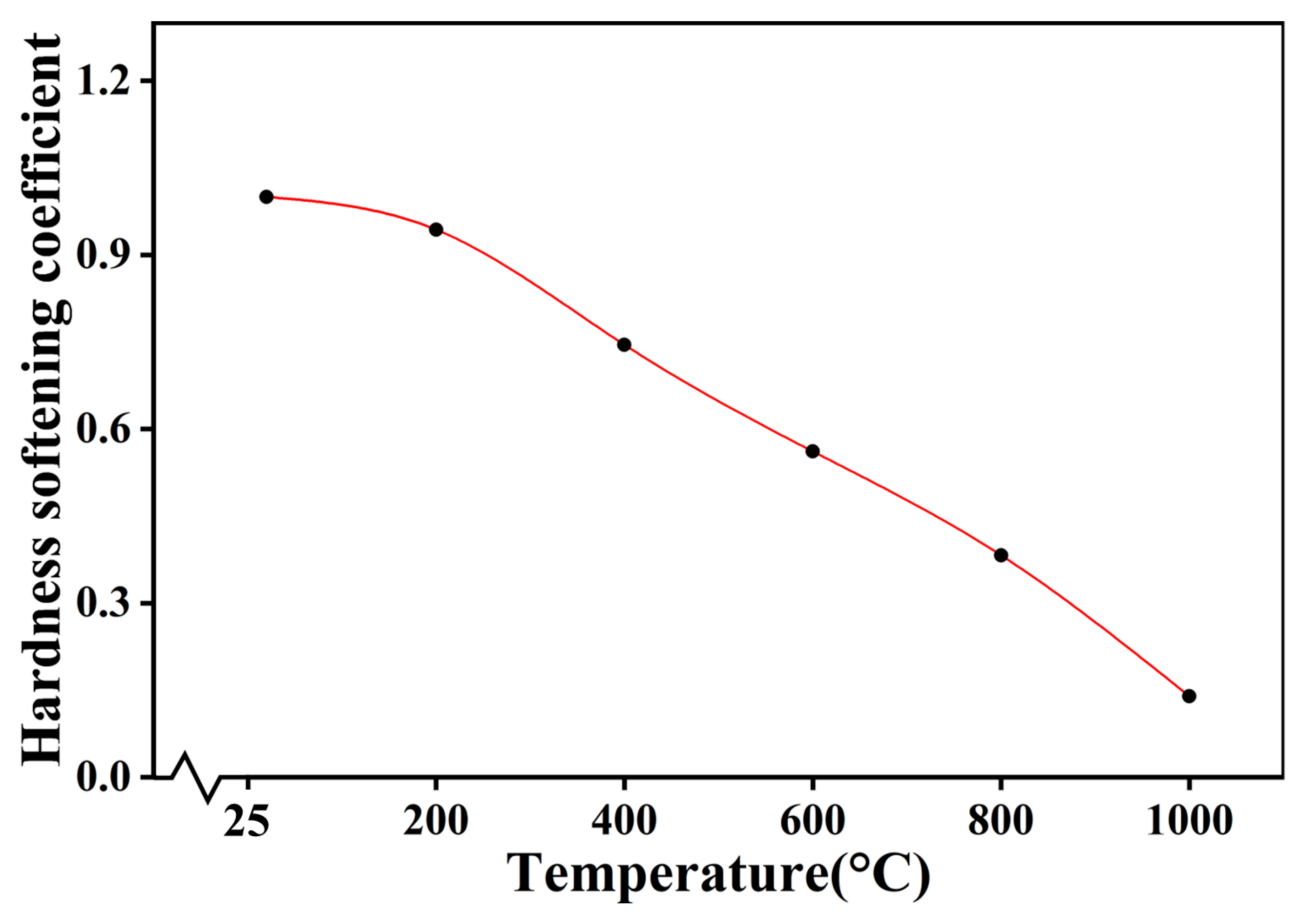
Hardness softening coefficient at different temperatures.

Due to the adiabatic temperature rise during the deformation process, the stress values at any given strain inherently represent the post-thermal softening values. In high-temperature SHPB impact tests, a coupled interaction exists between the strain rate term and the thermal softening term; directly employing the true stress–true strain curves to fit the constitutive model would inevitably compromise its predictive accuracy. To address this issue, high-temperature Vickers hardness tests were adopted in lieu of high-temperature SHPB impact tests to obtain the hardness softening coefficient. The true stress–true strain curves were subsequently transformed into isothermal stress–strain curves for constitutive fitting, thereby effectively eliminating the influence of the strain rate–temperature coupling. Moreover, since the deformation during high-temperature hardness testing occurs at the micrometer scale—which closely corresponds to the deformation scale prevalent in the cutting process—this approach enables a more accurate characterization of the thermal softening behavior of the material under cutting conditions. To further enhance the fidelity of the subsequent constitutive fitting, the actual true stress–true strain curves obtained at various strain rates were converted into their isothermal counterparts according to the conversion formula given in Equation (7), based on the temperature-dependent variation in the hardness softening coefficient. Figure 8 presents the hardness values of the quenched 42CrMo steel as a function of temperature. The static hardness softening coefficient of the material is defined as α. According to Equation (8), the temperature softening coefficient α_1_ for the quenched 42CrMo steel is obtained as expressed in Equation (9), and the hardness softening coefficients at different temperatures are shown in Figure 9. Consequently, the conversion of the actual stress–strain curves into isothermal curves based on the hardness softening coefficient can effectively eliminate the strain rate–temperature coupling effect and significantly improve the fitting accuracy of the constitutive model.

(7) $$\sigma_{i}=\sigma_{t}/\alpha$$

where *σ*_t_ represents the experimental (measured) stress value, *σ*_i_ denotes the isothermal stress value, and *α* is the temperature softening coefficient.

(8) $$\alpha =\frac{YD_{T}}{YD_{25}}$$

where *YD*_T_ is the hardness value measured at a given elevated temperature, and *YD*_25_ is the hardness value of the material at room temperature.

(9) $$\begin{array}{l} \alpha_{1}=-3.2977\times 10^{-17}T^{5}+9.8930\times 10^{-14}T^{4}-8.1126\times 10^{-10}T^{3}+2.1837\times 10^{-7}T^{2}\\ \;\;\;\;\;\;-1.0736\times 10^{-4}T+1.0021 \end{array}$$

**Figure 10:**
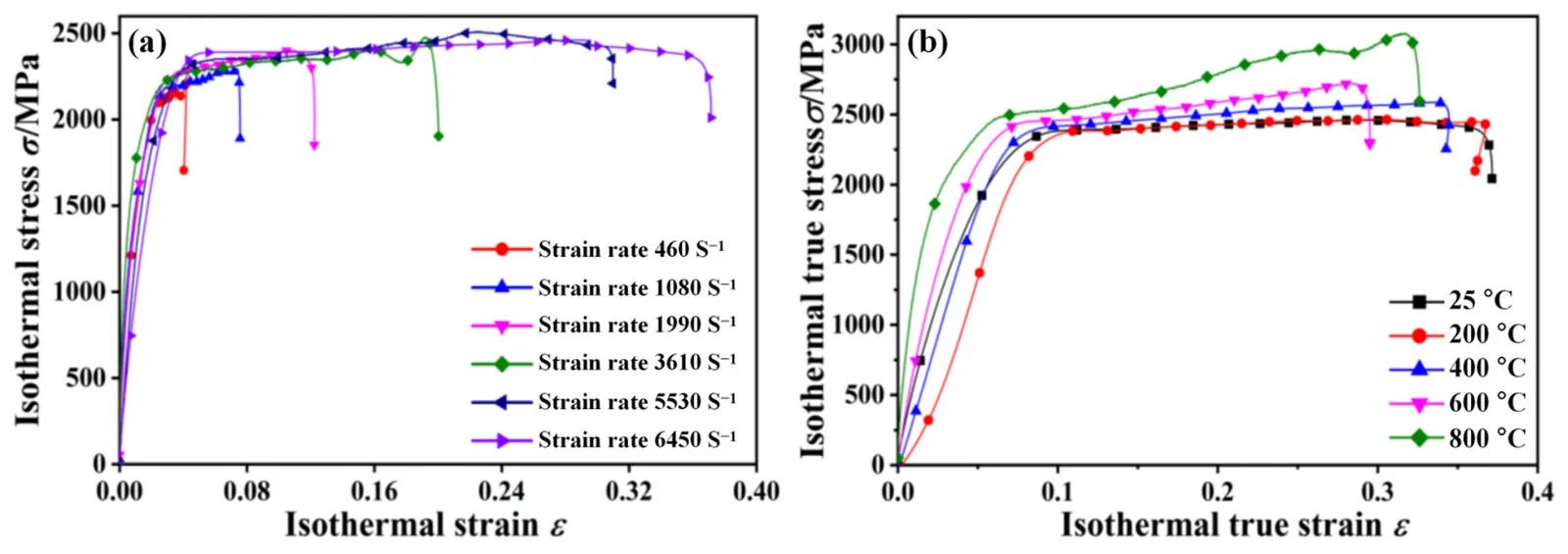
Isothermal stress–strain curves, with (**a**) showing those at different strain rates and (**b**) showing those at different temperatures.

According to Equations (7)–(9), the true stress–strain curves obtained from material tests at different temperatures were converted into the corresponding isothermal curves, with the results presented in Figure 10a,b.

### 3.4. The P-L Constitutive Model for Quenched 42CrMo Steel

The P-L constitutive model is mainly composed of three multiplicative terms, g(*ε*_s_), Γ(*έ*_s_), and Θ(*T*), as shown in Equation (10). These three terms reflect the strain hardening, strain rate hardening, and temperature softening characteristics of the material, respectively. This constitutive model assumes that the three characteristics of the material are independent of each other.

(10) $$\sigma \left(\varepsilon_{s},{\dot{\varepsilon }}_{s},T\right)=g\left(\varepsilon_{s}\right)*\Gamma \left({\dot{\varepsilon }}_{s}\right)*\Theta \left(T\right)$$

(11) $$g\left(\varepsilon_{s}\right)=\sigma_{0}{\left(1+\varepsilon_{s}/\varepsilon_{0}\right)}^{1/n}$$

(12) $$\Gamma \left({\dot{\varepsilon }}_{s}\right)={\left(1+{\dot{\varepsilon }}_{s}/{\dot{\varepsilon }}_{0}\right)}^{1/m}$$

(13) $$\Theta \left(T\right)=C_{0}+C_{1}T+C_{2}T^{2}+C_{3}T^{3}+C_{4}T^{4}+C_{5}T^{5}$$

(14) $$T=T_{0}+\Delta T$$

In Equations (10)–(14), *σ*_0_ is the yield strength (initial stress) at the reference temperature and reference strain rate (quasi-static condition); *ε*_0_ is the reference strain; *ε*_s_ is the plastic strain; *n* is the strain hardening coefficient; έ_s_ is the plastic strain rate; m is the strain rate hardening coefficient; *έ*_0_ is the reference strain rate (generally taken as 1 s^−1^); *C*_0_ is the thermal softening coefficient; *C*_1_,…,*C*_5_ are material parameters; *T*_0_ is the reference temperature (generally taken as room temperature); Δ*T* is the adiabatic temperature rise; and *T* is the total temperature.

- Determination of *σ*_0_ and *n*

Taking the isothermal stress–strain curve of quenched 42CrMo steel at a strain rate of 460 s^−1^ as the reference, it is assumed that the two terms reflecting the strain rate hardening and temperature softening characteristics of the material, Γ(*έ*_s_) and Θ(*T*), are both equal to 1. Thus, Equation (15) is obtained.

(15) $$\sigma (\varepsilon_{s})=g(\varepsilon_{s})=\sigma_{0}{\left(1+\frac{\varepsilon_{s}}{\varepsilon_{0}}\right)}^{1/n}$$

From Figure 10a in the main text, the material parameter *σ*_0_ of quenched 42CrMo steel is obtained as 1756.757 MPa. Taking the reference strain as 0.008 and taking the logarithm of both sides of Equation (15), Equation (16) is obtained.

(16) $$\ln\frac{\sigma (\varepsilon_{s})}{\sigma_{0}}=\frac{1}{n}\ln(1+\frac{\varepsilon_{s}}{\varepsilon_{0}})$$

**Figure 11:**
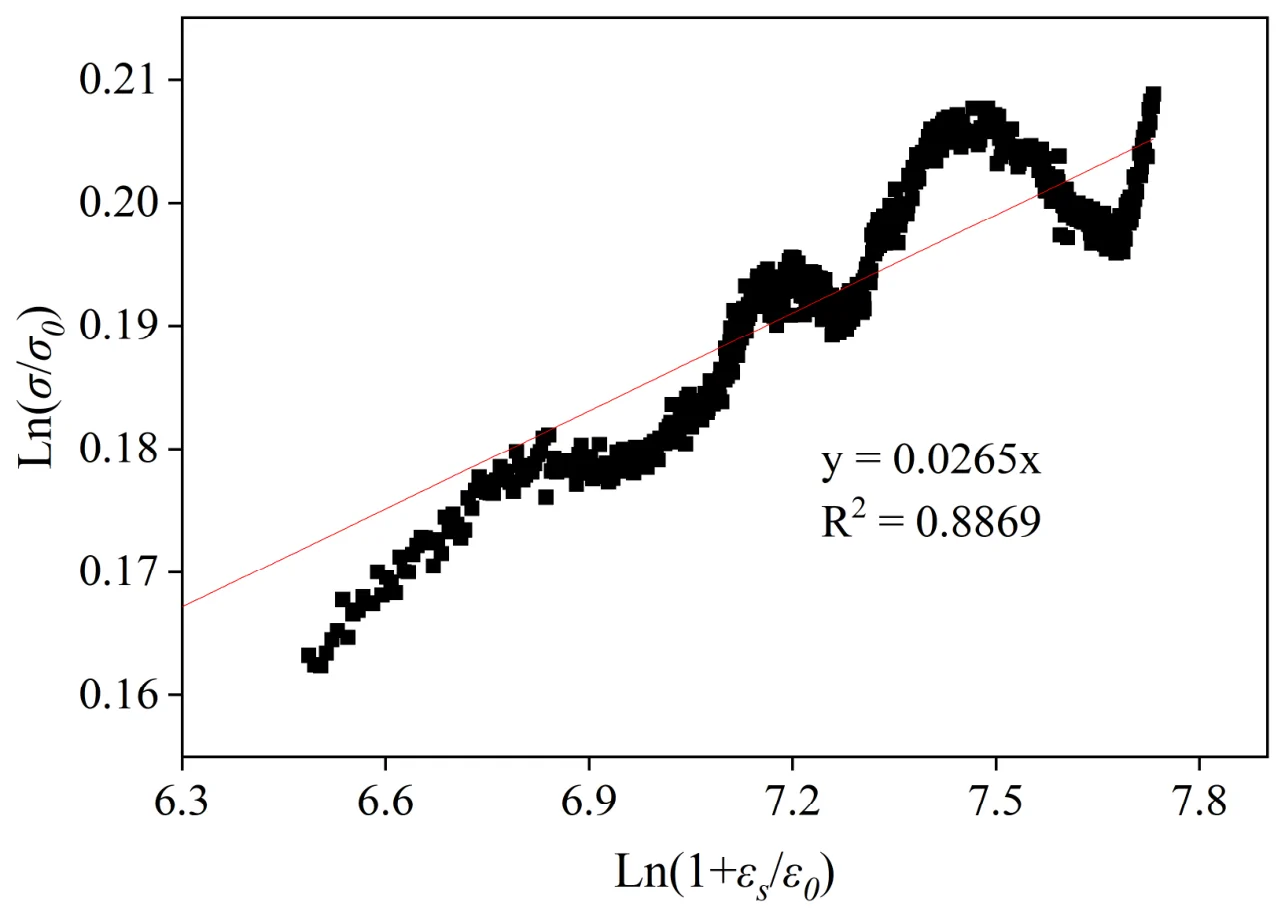
Relationship between ln(*σ*/*σ*_0_) and ln(1+*ε*_s_/*ε*_0_).

For quenched 42CrMo steel, the flow stress values corresponding to strains of 0.022–0.04 and the corresponding strains were substituted into Equation (16). Through linear fitting using the least squares method, the slope of the straight line (1/*n*) was obtained as 0.0265, and the material parameter *n* was further determined to be 37.736, as shown in Figure 11.

- Determination of *m*

The material parameter m was determined using the isothermal stress–strain curves over a strain rate range of 460–6450 s^−1^ at room temperature. When the material deforms at room temperature, it is assumed that the thermal softening term Θ(*T*) is equal to 1. Therefore, the constitutive model can be simplified to Equation (17).

(17) $$\sigma (\varepsilon_{s},\overset{•}{\varepsilon_{s}})=g(\varepsilon_{s}){\left(1+\frac{\overset{•}{\varepsilon_{s}}}{{\overset{•}{\varepsilon }}_{0}}\right)}^{1/m}$$

Taking the logarithm of both sides of Equation (17), Equation (18) is obtained.

(18) $$\ln\frac{\sigma (\varepsilon_{s},\overset{•}{\varepsilon_{s}})}{g(\varepsilon_{s})}=\frac{1}{m}\ln(1+\frac{\overset{•}{\varepsilon_{s}}}{{\overset{•}{\varepsilon }}_{0}})$$

**Figure 12:**
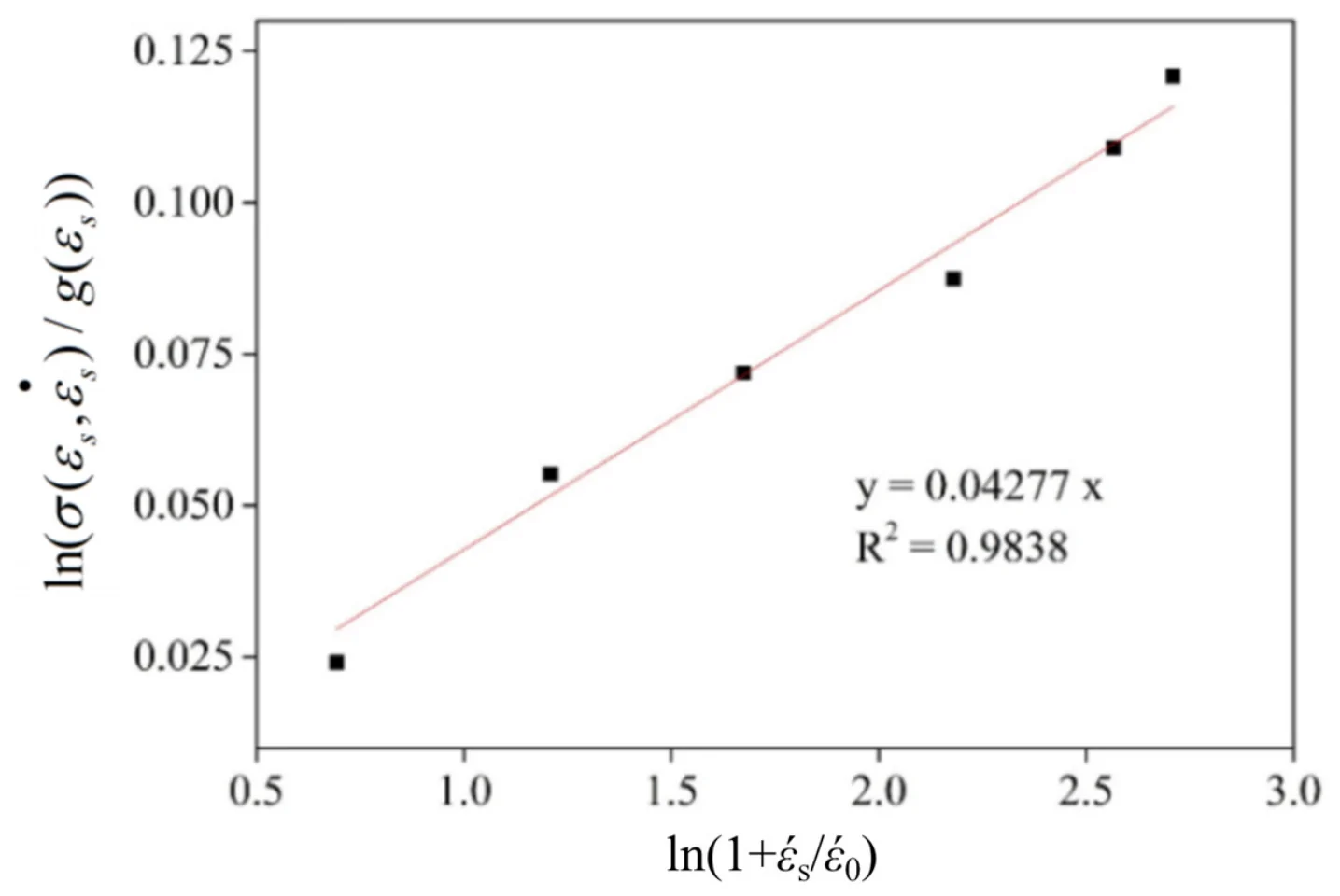
Relationship between ln(*ε*_s_,*έ*_s_)/g(*ε*_s_) and ln(1+*έ*_s_/*έ*_0_).

Since the adiabatic temperature rise is small at small strains, the strain softening effect is not significant. Therefore, the average values of the flow stress corresponding to different strain rates and the corresponding strain rates were substituted into Equation (18). Through linear fitting using the least squares method, the slope of the straight line (1/*m*) was obtained. Taking the reference strain rate as 460 s^−1^, the slope (1/*m*) was fitted to be 0.04277, and the material parameter m was further determined to be 23.381, as shown in Figure 12.

- Determination of thermal softening coefficients *C*_1_,…,*C*_5_

The material parameters *C*_1_,…,*C*_5_ were determined using the stress–strain curves over a strain rate range of 460–6450 s^−1^ and a temperature range of 25–800 °C. It is assumed that the strain hardening term g(*ε*_s_) and the strain rate hardening term Γ(*έ*_s_) are equal to 1. Therefore, the constitutive model can be simplified to Equation (19).

(19) $$\frac{\sigma \left(\varepsilon_{s},{\dot{\varepsilon }}_{s},T\right)}{g\left(\varepsilon_{s}\right)\Gamma \left({\dot{\varepsilon }}_{s}\right)}=\Theta \left(T\right)=C_{0}+C_{1}T+C_{2}T^{2}+C_{3}T^{3}+C_{4}T^{4}+C_{5}T^{5}$$

**Figure 13:**
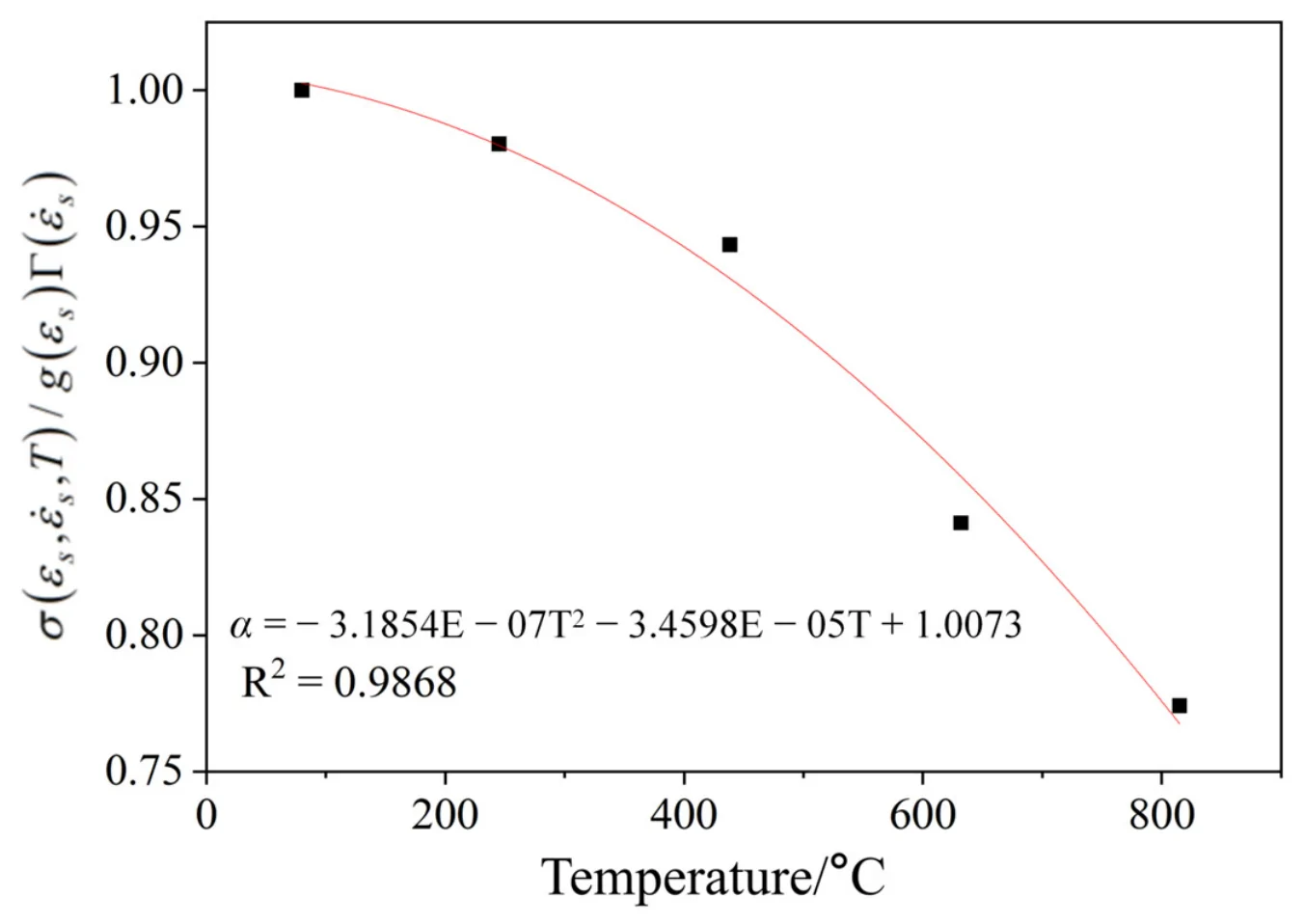
Relationship between *σ*(*ε*_s_,*έ*_s_,*T*)/(g(ε_s_)Γ(*έ*_s_)) and temperature.

By substituting the true stress at different temperatures into Equation (19) and performing polynomial fitting, the material parameters *C*_0_~*C*_5_ were obtained. The fitting curve is shown in Figure 13. For quenched 42CrMo steel, the material parameters *C*_0_~*C*_3_ are 1.0073, −3.4598 × 10^−5^ and −3.1854 × 10^−7^, respectively, while *C*_4_ and *C*_5_ are 0.

After determining the material parameters according to the above steps, the P-L constitutive model for quenched 42CrMo steel is as given by Equation (20).

(20) $$\begin{array}{l} \sigma (\varepsilon_{s},{\dot{\varepsilon }}_{s},T)=g(\varepsilon_{s})\Gamma ({\dot{\varepsilon }}_{s})\Theta (T)=1756.757\times {\left(1+\frac{\varepsilon_{s}}{0.00035}\right)}^{0.0265}{\left(\frac{{\dot{\varepsilon }}_{s}}{460}\right)}^{0.04277}\\ \times (1.0073-3.4598\times 10^{-5}T-3.1854\times 10^{-7}T^{2}) \end{array}$$

### 3.5. Calibration of J-C Constitutive Model for Quenched 42CrMo Steel

The general form of the J-C model is given by Equation (21).

(21) $$\sigma \left(\varepsilon_{s},{\dot{\varepsilon }}_{s},T\right)=g\left(\varepsilon_{s}\right)*\Gamma \left({\dot{\varepsilon }}_{s}\right)*\Theta \left(T\right)$$

(22) $$g\left(\varepsilon_{s}\right)=A+B{\left({\bar{\varepsilon }}^{p}\right)}^{n}$$

(23) $$\Gamma \left({\dot{\varepsilon }}_{s}\right)=1+C\ln{\dot{\varepsilon }}^{*}$$

(24) $$\Theta \left(T\right)=1-{\left(T^{*}\right)}^{m}$$

(25) $$\bar{\sigma }=(A+B{\left({\bar{\varepsilon }}^{p}\right)}^{n})\times (1+C\ln{\dot{\varepsilon }}^{*})\times (1-{\left(T^{*}\right)}^{m})$$

(26) $$T^{*}=\left(T-T_{r}\right)/\left(T_{m}-T_{r}\right)$$

In Equations (21)–(26), *σ* is the yield stress of the material; *A* is the yield strength at the reference temperature and reference strain rate; *B* is the strain hardening coefficient; *n* is the strain hardening exponent; *C* is the strain rate hardening coefficient; *m* is the thermal softening exponent; $\bar{\varepsilon }$_p_ is the equivalent plastic strain; $\bar{\varepsilon }$_p_ = *ε*_p_/*ε*_0_; *ε*_p_ is the plastic strain; *ε*_0_ is the reference strain; έ* is the dimensionless plastic strain rate; έ* = *έ*/*έ*_0_; *έ*_0_ is the reference strain rate (generally taken as 1 s^−1^); *έ* is the plastic strain rate; T* is the homologous temperature; *T*_r_ is the reference temperature, generally taken as room temperature; *T*_m_ is the melting point of the material; and *T* is the instantaneous temperature.

- Determination of *A*, *B*, and *n*

First, assuming that the strain rate hardening term and the temperature softening term are equal to 1, the J-C constitutive model can be simplified to Equation (27).

(27) $$\bar{\sigma }=A+B{\bar{\varepsilon }}^{pn}$$

The work hardening constants of the material were determined according to Figure 10a in the main text. The material parameter σ0 was found to be 1756.757 MPa, and the reference strain was taken as 0.015. According to Equation (27), *A* = *σ*_0_ = 1756.757. By rearranging the terms and taking the logarithm of both sides of the equation, Equation (28) is obtained.

(28) $$\ln(\bar{\sigma }-A)=\ln B+n\ln({\bar{\varepsilon }}^{p})$$

**Figure 14:**
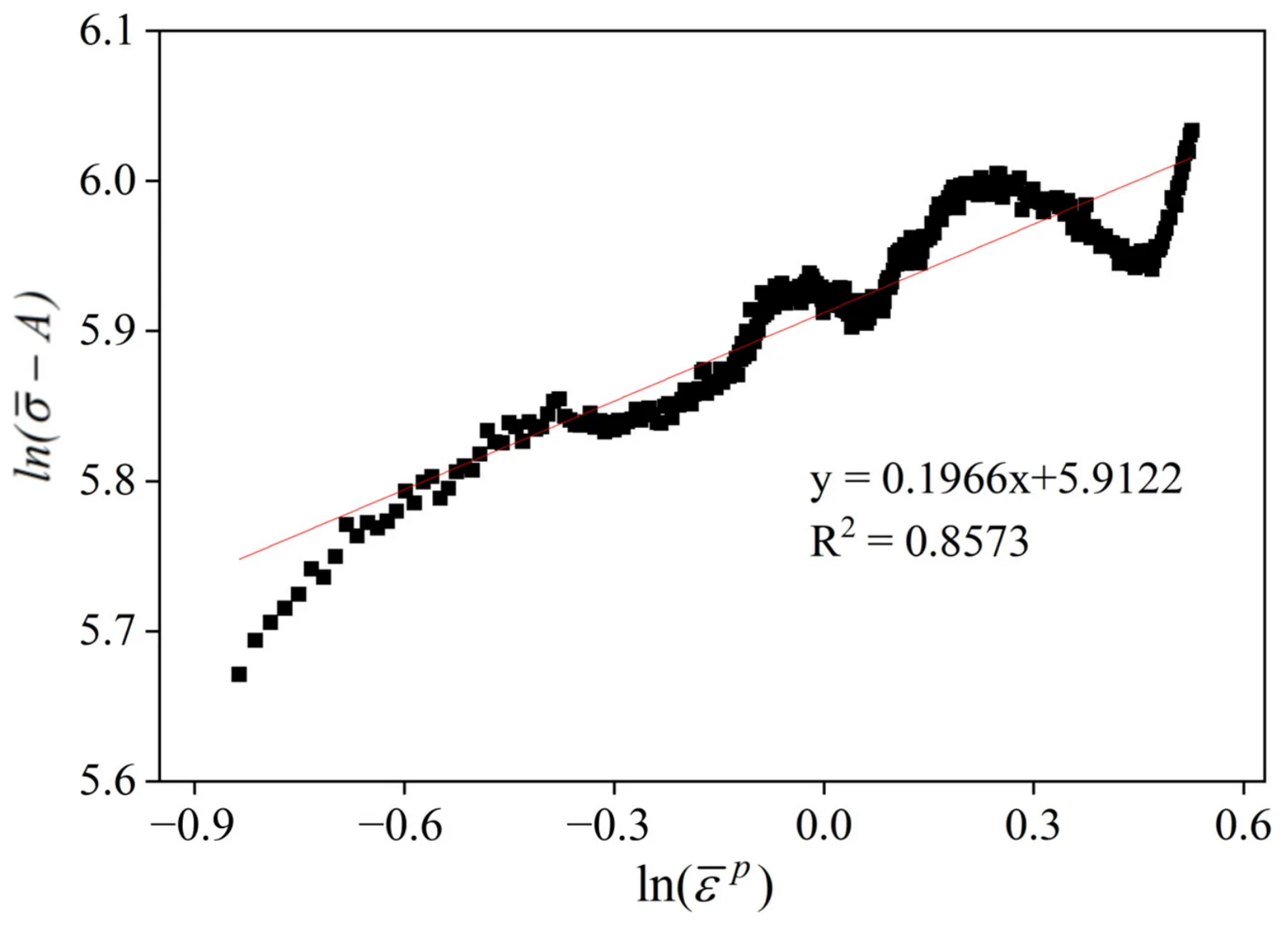
Relationship between ln($\bar{\sigma }$ − *A*) and ln($\bar{\varepsilon }$_p_).

The slope of the fitted curve obtained from the relationship between ln($\bar{\sigma }$ − *A*) and ln($\bar{\varepsilon }$_p_) in Figure 14 is *n*. For quenched 42CrMo steel, *n* = 0.1966 and *B* = 369.518 were obtained.

- Determination of *C*

Assuming that the temperature softening term is equal to 1, the J-C constitutive model can be simplified to Equations (29) and (30).

(29) $$\bar{\sigma }=(A+B{\bar{\varepsilon }}^{pn})(1+C\ln\frac{{\dot{\varepsilon }}^{*}}{\varepsilon_{0}})$$

(30) $$\frac{\bar{\sigma }}{g(\varepsilon )}=1+C\ln\frac{{\dot{\varepsilon }}^{*}}{\varepsilon_{0}}$$

**Figure 15:**
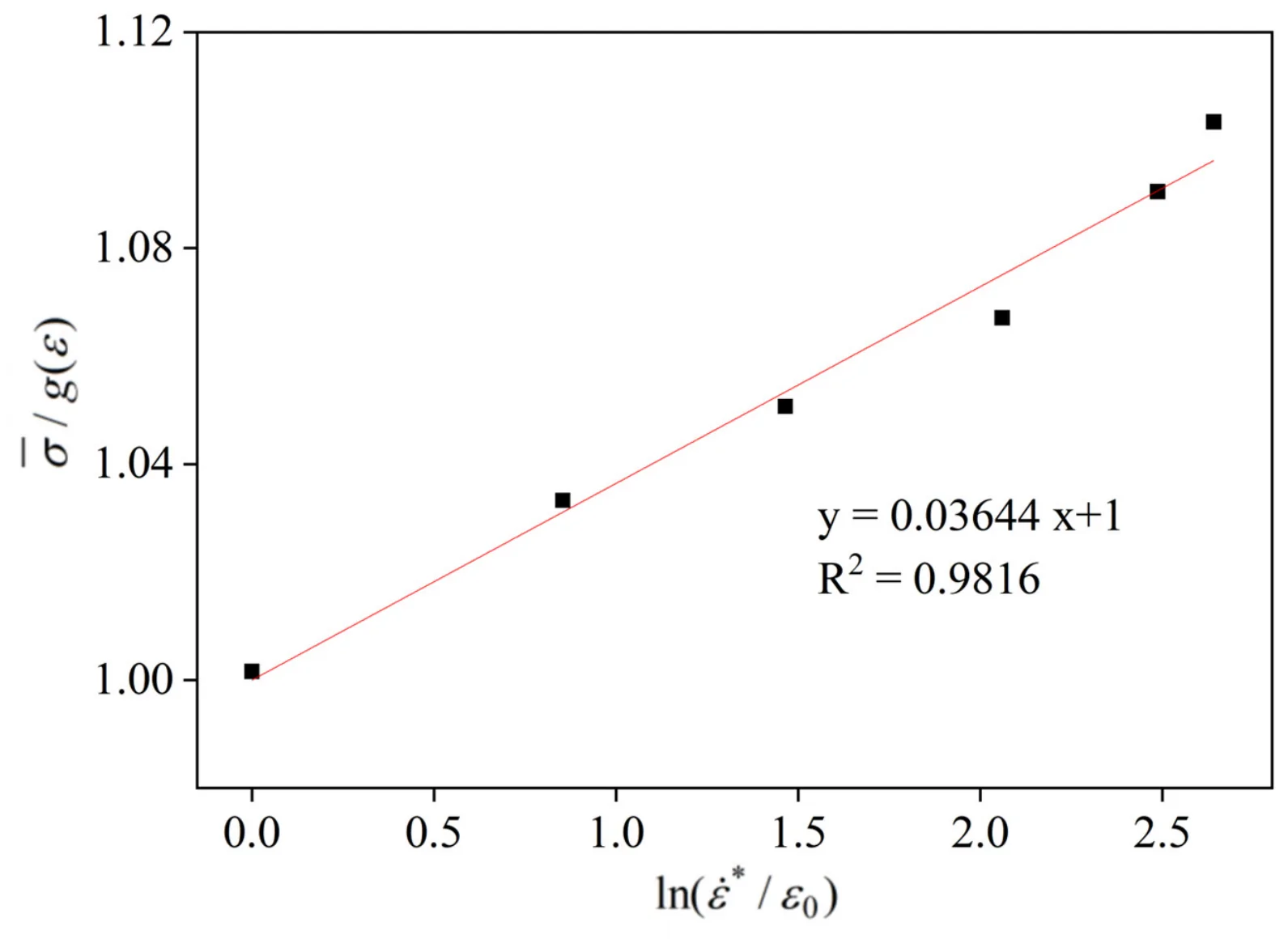
Relationship between $\bar{\sigma }/g(\varepsilon )$ and $\ln({\dot{\varepsilon }}^{*}/\varepsilon_{0})$.

As shown in Figure 15, the linear fitting of the curve yields *C* = 0.03644.

- Determination of *m*

The temperature softening term is fitted according to Equation (31).

(31) $$\frac{\bar{\sigma }}{g\left(\varepsilon_{s}\right)*\Gamma \left({\dot{\varepsilon }}_{s}\right)}=1-{\left(T^{*}\right)}^{m}$$

Taking the logarithm of both sides of Equation (31) and rearranging, Equation (32) is obtained.

(32) $$\ln(1-\frac{\bar{\sigma }}{g\left(\varepsilon_{s}\right)*\Gamma \left({\dot{\varepsilon }}_{s}\right)})=m\ln(T^{*})$$

**Figure 16:**
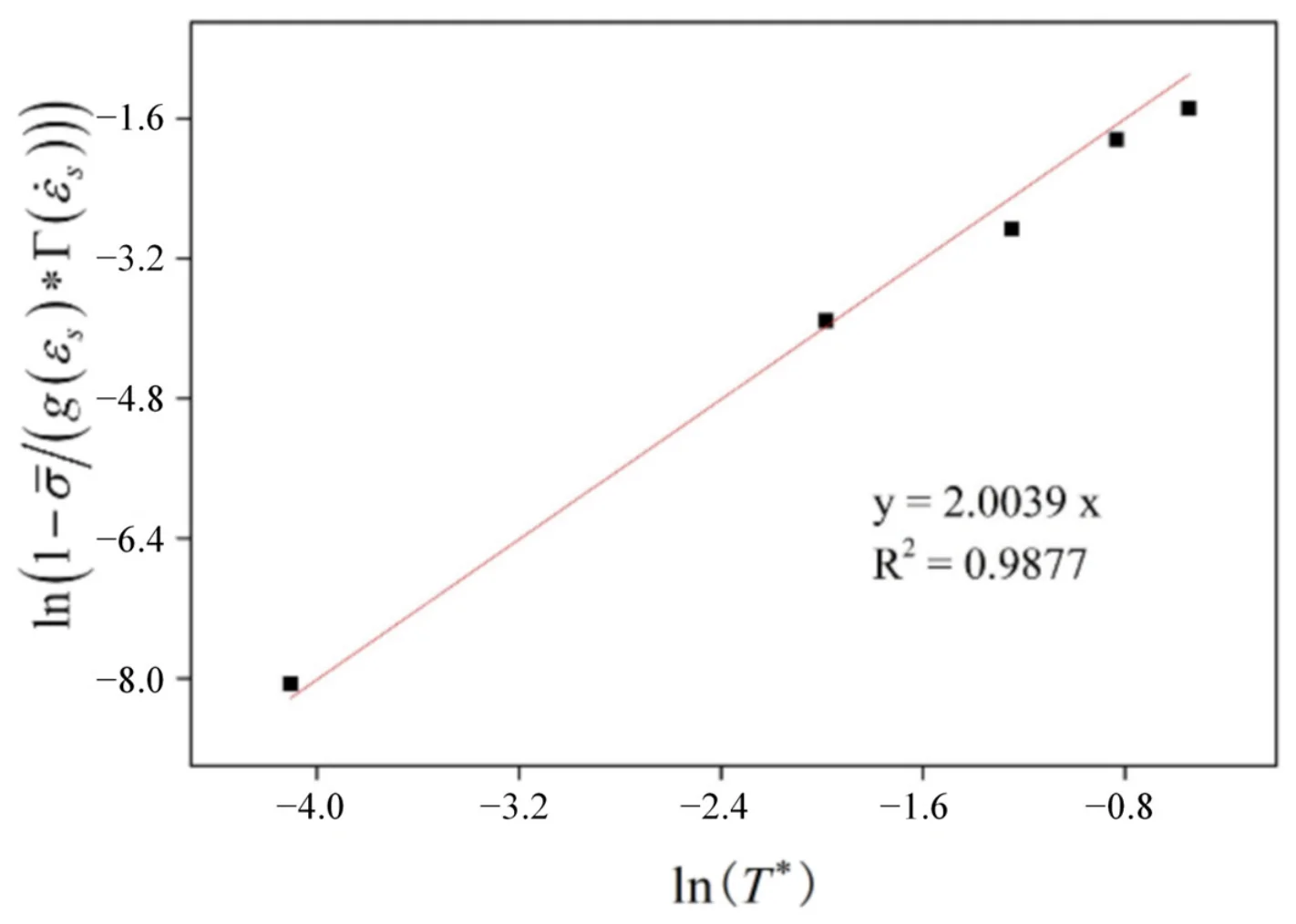
Relationship between $\ln(1-\frac{\bar{\sigma }}{g\left(\varepsilon_{s}\right)*\Gamma \left({\dot{\varepsilon }}_{s}\right)})$ and $\ln(T^{*})$.

Finally, the temperature softening term of the material constitutive model was determined according to the stress–strain curves of the material at different temperatures in Figure 10b of the main text. The fitting result is shown in Figure 16. For quenched 42CrMo steel, *m* = 2.0039 was obtained.

After determining the material parameters according to the above steps, the J-C constitutive model for quenched 42CrMo steel is given by Equation (33).

(33) $$\bar{\sigma }=(1756.757+369.518{\left(\frac{\varepsilon^{P}}{0.015}\right)}^{0.1966})(1+3.64\times 10^{-2}\ln(\frac{\dot{\varepsilon }}{460}))(1-(\frac{T-25}{1400})2.0039)$$

### 3.6. Statistical Comparison Between the P-L and J-Cook Constitutive Models for Quenched 42CrMo Steel

**Figure 17:**
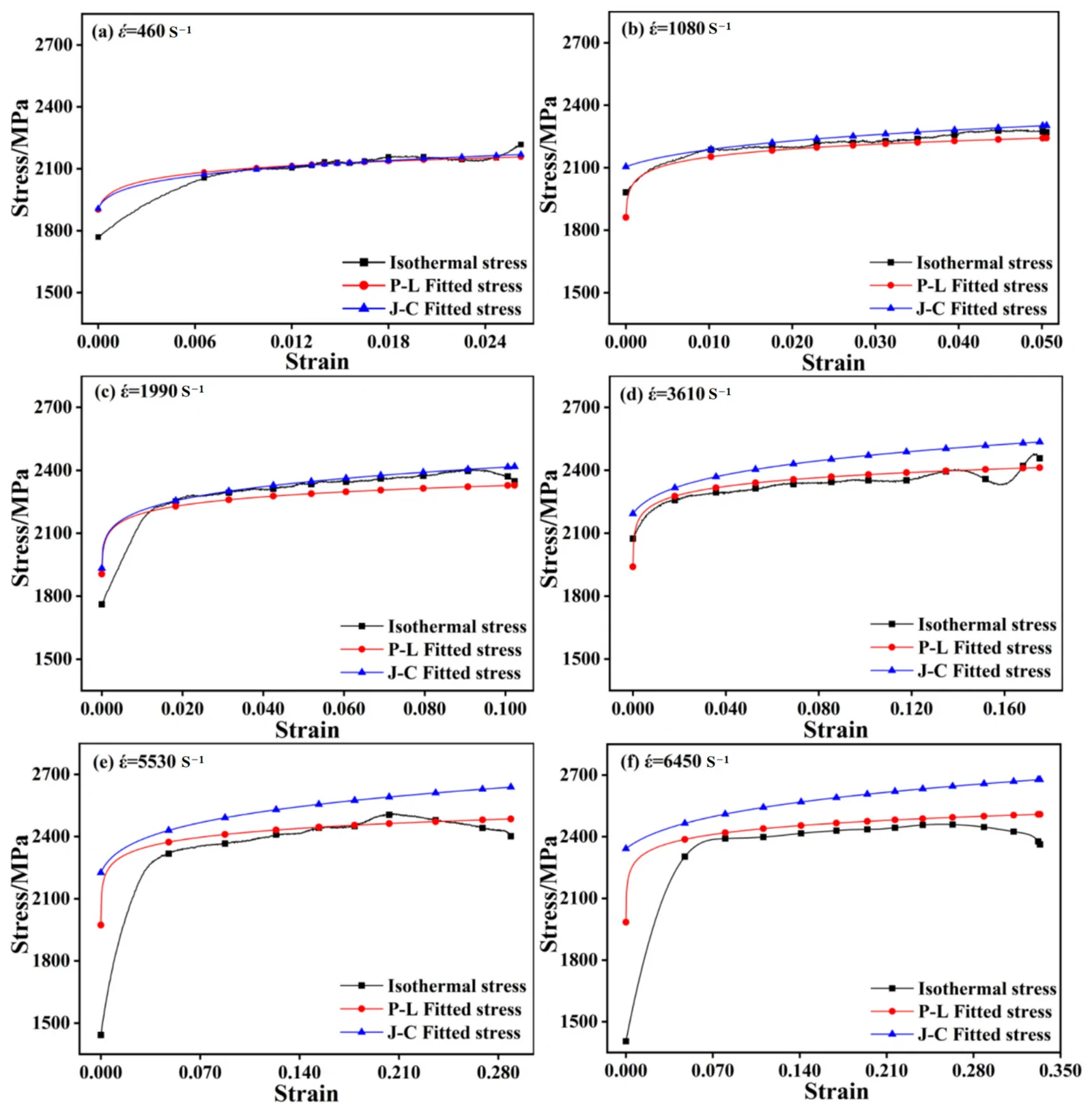
Comparison between measured isothermal results and constitutive model fitting at different strain rates (*T* = 25 °C).

**Figure 18:**
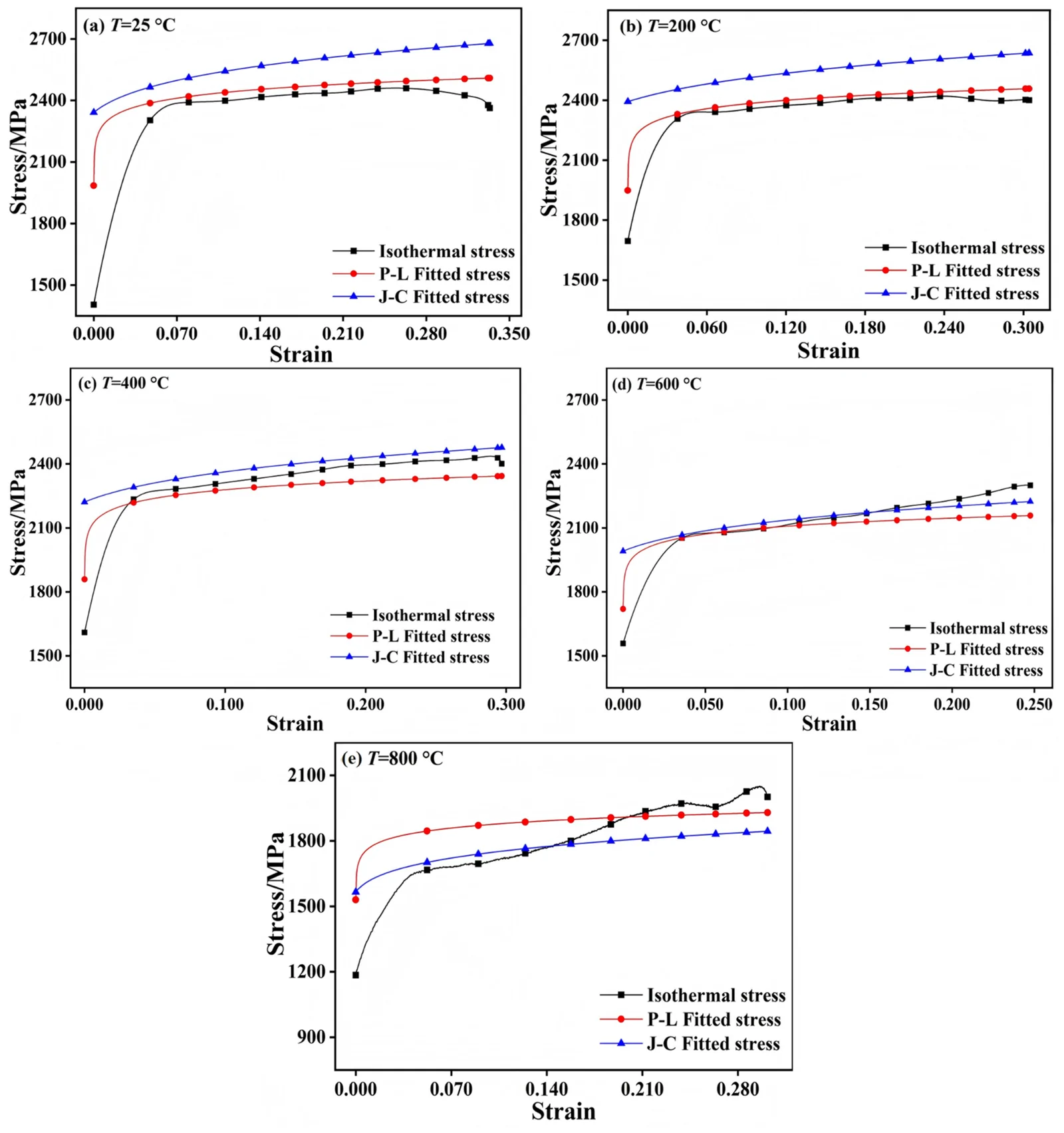
Comparison between measured isothermal results and constitutive model fitting at different temperatures (έ = 6450 s^−1^).

The fitting results of the material constitutive equations for quenched 42CrMo steel based on the P-L and J-C constitutive models were compared with the experimental results. Figure 17 shows the comparison between the model fitting curves and the experimental results for quenched 42CrMo steel at different strain rates, and Figure 18 shows the comparison at different temperatures. It can be seen from the comparison that both established constitutive equations can reasonably reflect the dynamic mechanical properties of the material. The error of the model fitting results was calculated using Equation (34), and the fitting accuracy of the two models was compared in terms of relative error. The results are shown in Table 4. As can be seen from Table 4, the fitting accuracy of the P-L constitutive model is higher than that of the J-C constitutive model. Therefore, the P-L constitutive model is more suitable for quenched 42CrMo steel.

(34) $$e_{r}=\frac{\left|\sigma_{c}-\sigma_{m}\right|}{\sigma_{c}}\times 100\%$$

where *σ*_c_ and *σ*_m_ are the measured and predicted flow stress values, respectively, and *e_r_* is the error.

## 4. Conclusions

1. The flow stress of quenched 42CrMo steel under high strain rate loading exhibits significant strain hardening and temperature softening effects, while its strain rate sensitivity is relatively low. The strain rate sensitivity coefficient is 34.707 in the strain rate range of 460–1990 s^−1^ and decreases to 7.214 in the range of 1990–6450 s^−1^. Temperature sensitivity increases with increasing temperature, with a sensitivity coefficient of 0.361 MPa/°C in the temperature range of 25–400 °C, and increases to 0.685 MPa/°C in the range of 400–800 °C.
2. The adiabatic temperature rise increases with increasing strain rate and decreases with increasing initial temperature. High-temperature environments can partially suppress the adiabatic temperature rise effect, reducing both the magnitude of the temperature rise and its rate of increase.
3. The isothermal stress–strain curves constructed based on the hardness softening coefficient can effectively decouple the coupling effect between strain rate and temperature. Both the P-L model and the J-C model can describe the dynamic mechanical behavior of quenched 42CrMo steel, but the P-L model shows closer agreement with the experimental data. The average fitting error of the P-L model is 1.98%, which is significantly lower than that of the J-C model (3.23%), and it shows better fitting stability at a strain rate of 6450 s^−1^ and a temperature of 800 °C.

## Figures and Tables

**Table 1:** Chemical composition of 42CrMo steel (wt.%).

**Composition**	C	Si	Mn	P	S	Ni	Cr	Mo	Cu	V	Fe
**Content**	0.41	0.24	0.74	0.012	0.023	0.03	1.15	0.21	0.08	0.01	Bal.

**Table 2:** Basic mechanical properties of 42CrMo steel.

Yield Strength/MPa	Tensile Strength/MPa	Elongation Rate	Impact Energy/J	Hardness/HV
1352 ± 21.7	1770 ± 15.2	11.6 ± 0.3	41.0 ± 1.6	596.8 ± 7.6

**Table 3:** Experimental scheme for Hopkinson pressure bar tests.

Impact Pressure/MPa	Strain Rate/s^−1^	Temperature/°C
0.05	460	25
0.075	1080	
0.10	1990	
0.15	3610	
0.20	5530	
0.25	6450	
0.25	6450	200
		400
		600
		800

Note: The impact pressure is 0.25 MPa. The actual measured strain rates range from approximately 6372.4 s^−1^ to 6531.2 s^−1^; however, a simplified value of 6450 s^−1^ is used in the table for clarity. This simplification has no significant impact on the primary findings.

**Table 4:** Error comparison of constitutive model fitting results for quenched 42CrMo steel.

Strain Rate $\dot{\varepsilon }$/s^−1^	Temperature *T*/°C	Strain *ε*	Stress *σ*_c_/MPa	P-L Model *σ*_c_/MPa	J-C Model *σ*_c_/MPa	P-L Model Error/%	J-C Model Error/%
460	25	0.01	2105.11	2105.75	2099.24	0.03%	0.28%
		0.015	2128.35	2127.22	2127.06	0.05%	0.06%
		0.02	2155.03	2142.82	2148.48	0.57%	0.30%
1080	25	0.01	2187.16	2151.79	2187.21	1.62%	0.00%
		0.03	2223.89	2212.90	2258.81	0.49%	1.57%
		0.05	2274.71	2241.99	2301.43	1.44%	1.17%
1990	25	0.02	2272.04	2234.27	2262.71	1.66%	0.41%
		0.06	2347.43	2297.68	2361.68	2.12%	0.61%
		0.1	2371.93	2327.88	2415.62	1.86%	1.84%
3610	25	0.05	2306.92	2337.27	2399.56	1.32%	4.02%
		0.1	2350.83	2379.05	2469.87	1.20%	5.06%
		0.15	2369.33	2403.79	2516.40	1.45%	6.21%
5530	25	0.05	2328.63	2376.39	2434.52	2.05%	4.55%
		0.15	2435.32	2443.93	2552.90	0.35%	4.83%
		0.25	2466.60	2476.03	2618.66	0.38%	6.17%
6450	25	0.05	2321.62	2390.68	2469.63	2.97%	6.38%
		0.15	2421.74	2458.83	2576.37	1.53%	6.39%
		0.25	2460.60	2491.21	2639.24	1.24%	7.26%
6450	200	0.05	2339.48	2346.46	2469.84	0.30%	5.57%
		0.15	2388.02	2413.40	2556.43	1.06%	7.05%
		0.25	2416.11	2445.17	2612.25	1.20%	8.12%
6450	400	0.05	2274.00	2239.51	2311.85	1.52%	1.66%
		0.15	2355.19	2303.12	2401.21	2.21%	1.95%
		0.25	2416.33	2333.39	2456.53	3.43%	1.66%
6450	600	0.05	2075.93	2071.64	2087.11	0.21%	0.54%
		0.15	2171.02	2130.46	2173.19	1.87%	0.10%
		0.25	2300.09	2157.94	2223.94	6.18%	3.31%
6450	800	0.05	1662.78	1843.37	1699.21	10.86%	2.19%
		0.15	1788.95	1895.71	1780.57	5.97%	0.47%
		0.25	1966.10	1920.57	1826.05	2.32%	7.12%
Average Error						1.98%	3.23%
Mean Absolute Error (MAE)						42.37 MPa	75.05 MPa
Root Mean Square Error (RMSE)						58.06 MPa	98.39 MPa

## Data Availability

The original contributions presented in this study are included in the article. Further inquiries can be directed to the corresponding authors.
